# Reviving a Smile: A Multidisciplinary Approach to Dentin Dysplasia

**DOI:** 10.7759/cureus.59697

**Published:** 2024-05-05

**Authors:** Arun Vignesh KR, Krishnakumar Raja, Raj Kumar Krishnan, Pavithra Vijayakumar, Arul Prakash Kalaimani

**Affiliations:** 1 Oral and Maxillofacial Surgery, SRM Dental College and Hospitals, Chennai, IND; 2 Oral and Maxillofacial Surgery, SRM Dental College, Chennai, IND; 3 Oral and Maxillofacial Pathology, SRM Dental College, Chennai, IND

**Keywords:** dentin dysplasia, dspp, implant, esthetics, familial

## Abstract

Dentin dysplasia Type 1 (DD1) is an uncommon inherited condition marked by structural irregularities in dentin, leading to notable dental abnormalities. Clinically, patients typically present with generalized slight yellowish discoloration and tooth mobility, while radiographic examination often reveals a reduced pulp chamber with the absence of pulp stones, a hallmark feature of DD1. Treatment involves a multidisciplinary approach including extraction of affected teeth, direct sinus lift procedure bilaterally, implant placement, and subsequent fixed prosthesis placement. In a recent case, after six months, a patient demonstrated improved oral health-related quality of life with stabilized implant-supported prostheses providing functional and esthetic benefits. This emphasizes the importance of early diagnosis and intervention in managing DD1, underscoring the effectiveness of a multidisciplinary approach in enhancing oral function and esthetics. Further research is warranted to deepen our understanding of the genetic basis of this condition and develop targeted therapies.

## Introduction

Dysplasia refers to the abnormal development or growth of cells, tissues, or organs, often leading to functional impairments. Dentin dysplasia, a rare hereditary disorder typically inherited in an autosomal dominant manner, specifically affects the growth and structure of dentin within teeth [1]. This condition, characterized by abnormal dentin formation, presents numerous oral health challenges and implications. Dentin dysplasia manifests in two primary types: Type I and Type II, each with distinct characteristics and symptoms. Dentin dysplasia presents in two primary types: Type I, also known as radicular, and Type II, referred to as coronal. Type I is the more prevalent variant, with both types potentially affecting single or multiple teeth across primary and permanent dentition. Additionally, literature has described combinations of both types. Specifically, four distinct forms of Type I and one form of Type II have been identified. Both types affect both primary and permanent dentitions and can result in prematurely exfoliated and highly mobile teeth due to underdeveloped root structures. The condition arises from mutations in the dentin sialophosphoprotein gene (DSPP), located on chromosome 4q13-21, which encodes proteins crucial for dentinogenesis [2].

In the present case, the patient presented with generalized slight yellowish discoloration and tooth mobility upon clinical examination. Radiographic assessment unveiled a characteristic feature of dentin dysplasia Type I (DD1), with a reduction in pulp chamber size and the absence of pulp stones [3]. These findings align with the classic clinical and radiographic manifestations associated with DD1, emphasizing the importance of early recognition and intervention in managing this rare hereditary disorder.

## Case presentation

A 21-year-old female patient presented to our department seeking consultation for loose teeth and expressing interest in undergoing braces treatment. Notably, her father had previously experienced a similar condition, which led to the need for a removable prosthesis due to complete tooth loss in his jaws. Upon thorough intraoral examination, her teeth appeared to be of normal size and shape but exhibited generalized grade III mobility, a reduced vertical dimension, and significant malocclusion, compounded by the absence of teeth 31 and 41.

A panoramic X-ray was obtained, revealing features consistent with DD1. While the crowns of the affected teeth appeared normal, there were evident abnormalities in root development. Specifically, the pulp chambers and root canals displayed irregular shapes and diameters, with a notable absence or reduction in dentinal tubules. These structural alterations led to the radiographic appearance of shortened, blunted roots and obliterated pulp chambers. In addition to the clinical and radiographic findings, comprehensive hematological and biochemical investigations were conducted, with all results falling within normal ranges. Considering the radiographic evidence, a range of potential differential diagnoses were considered, including dentinogenesis imperfecta, amelogenesis imperfecta, Ehlers-Danlos syndrome, and regional odontodysplasia. A final diagnosis of dentin dysplasia was made.

Treating dentin dysplasia presents numerous challenges for dentists. Patients with this condition often experience early tooth loss, misalignment, mobility, and insufficient bone support. Performing endodontic treatment on affected teeth is typically not feasible due to the intricate root canals or the extensively altered internal pulp structure. While orthodontic intervention may be recommended, it must be approached cautiously. Short roots resistant to orthodontic forces can lead to further root resorption, tooth looseness, and premature tooth loss.

**Figure 1:**
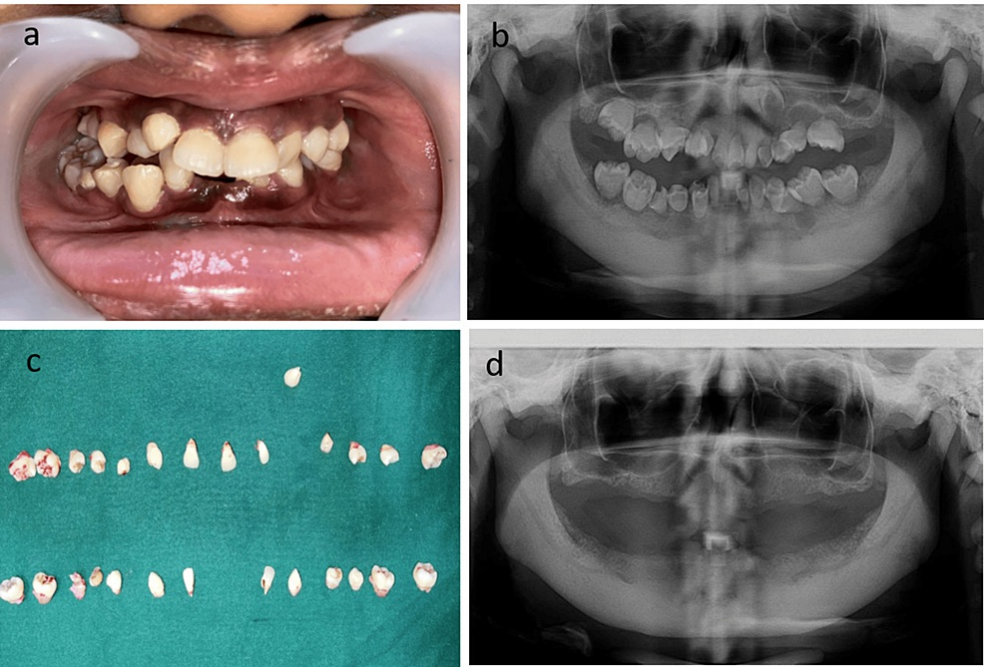
a) Intraoral photograph showing dentition with dentin dysplasia. b) Pre-operative orthopantomogram showing the rootless teeth appearance of all teeth. c) Extraction of all teeth performed. d) Post-extraction radiograph showing complete edentulous space

**Figure 2:**
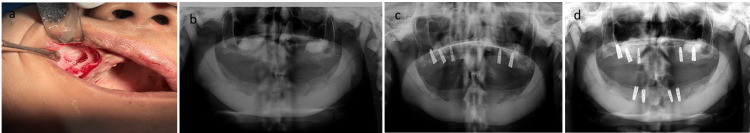
a) Lateral window direct sinus lift technique or the lateral approach was made. b) Orthopantomogram showing the lateral window. c) and d) Implant placement done

Considering the clinical and radiographic examination (Figures 1a, 1b), extraction of all teeth was initiated and performed under local anesthesia using lignocaine with adrenaline (Figures 1c, 1d). To reconstitute the resorbed bone in the maxilla, a bilateral direct sinus lifting procedure with a Bio-OSS bone graft was done (Figure 2a). Post-operative healing was uneventful and no dehiscence defect occurred (Figure 2b). After six months of healing, surgical implant placement (Dentium implants) was performed under conscious sedation (Figure 2c, 2d). The mucoperiosteal flap was elevated, and the dental implants were inserted in the maxilla and mandible, respectively. The patient was provided with a temporary prosthesis for two months.

**Figure 3:**
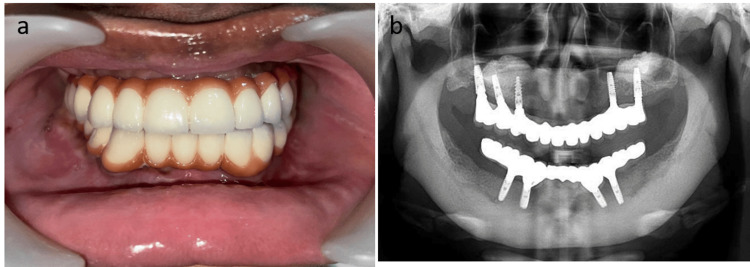
a) Intraoral image showing done implant prosthesis. b) Six months postoperative radiograph of the implant placement

After four months of healing, a stage 2 procedure was performed and the implants were uncovered and a healing abutment was placed. All the implants were osseointegrated with the new bone (22). Using the open tray impression technique, the final restoration was fabricated with various jig trials and metal trials. Final implant prosthesis loading was done (Figure 3a, 3b).

**Figure 4:**
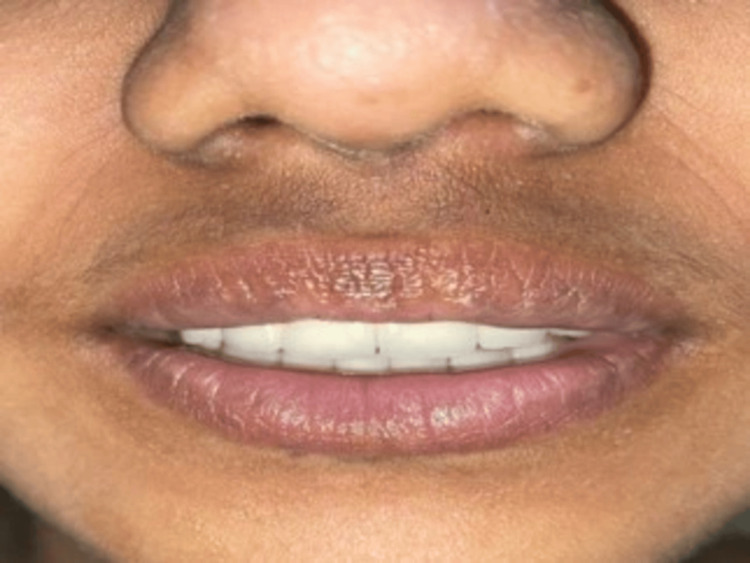
Post-treatment image indicating successful smile design interventions

A noticeable improvement in smile aesthetics and heightened patient satisfaction was observed post-treatment, indicating successful smile design interventions (Figure 4).

In the realm of healthcare, diagnostic errors are recognized widely, yet the dental field often lacks thorough data on such occurrences. Nevertheless, it is imperative to report instances of misdiagnoses and diagnostic errors in dentistry. A case study, like the one involving the father, underscores the critical need to address these shortcomings. Beyond simply missing a diagnosis, this case exposes broader failures within dental practice. Sometimes, dental professionals may demonstrate excessive confidence and superficiality in patient care, underscoring the significance of identifying and correcting diagnostic errors in dentistry.

## Discussion

Dentin dysplasia is a rare hereditary disorder characterized by abnormal dentin formation, impacting tooth growth and structure. It manifests in two types: Type I and Type II, each with distinct symptoms [4]. Type I, also known as radicular dentin dysplasia, is marked by defective dentin development, resulting in clinically normal-looking teeth that are highly mobile and prone to premature exfoliation due to underdeveloped root structures. Type II's incidence and prevalence remain unknown, but it affects both primary and permanent dentitions. Dentin dysplasia stems from mutations in the DSPP on chromosome 4q13-21, disrupting dentinogenesis [5].

DD1 primarily affects the roots of teeth, resulting in short, blunted roots with defective dentin that may not mineralize properly. This can lead to weakened teeth prone to fractures and premature loss. Some individuals may also experience early tooth eruption or alignment issues [6]. In contrast, Type II predominantly affects the crown of the tooth and is inherited in an autosomal recessive manner. The characteristic feature of Type II is the presence of a transparent or opalescent appearance, giving the teeth a "shell-like" look. Additionally, teeth affected by Type II may exhibit discoloration and heightened susceptibility to decay and enamel loss [7].

Histopathological examination reveals distinct abnormalities in dentin structure and composition, varying between the two types. Type I presents with irregularly shaped pulp chambers and root canals, often with reduced or absent dentinal tubules, resulting in short, blunt roots and obliterated pulp chambers on radiographs [8]. Meanwhile, Type II shows areas of opalescence or translucency due to irregular mineralization, along with signs of inflammation or fibrosis in the pulpal tissues. Notably, interglobular dentin, characterized by non- or hypo-mineralized areas between globular dentin, is a hallmark of Type II dentin dysplasia [8,9].

This familial case highlights the potential challenges in diagnosing DD1 when radiographic indicators are absent or inconclusive. DDI, an autosomal dominant hereditary disorder impacting dentin formation, is typified by symptoms such as tooth mobility and discomfort linked to spontaneous dental abscesses or cysts. 

In DD1, both primary and permanent dentitions are affected, despite the teeth appearing clinically normal in shape [10]. However, upon radiographic examination, abnormalities in dentin formation and pulp obliteration become apparent. Type I can be further classified into four subtypes: Subtype 1a, characterized by the absence of a pulp chamber and root formation, often accompanied by frequent peri-radicular radiolucencies; Subtype 1b, exhibits a single small, horizontally oriented, crescent-shaped pulp with short roots and frequent peri-apical radiolucencies; Subtype 1c, displays two horizontal or vertical, crescent-shaped pulpal remnants surrounding a central dentine island, along with shortened root length and variable peri-apical radiolucencies; Subtype 1d, features a visible pulp chamber and canal, near-normal root length, large pulp stones in the coronal portion of the canal, root constriction apical to the stone, and few peri-apical radiolucencies [11].

In contrast, DD2 is distinguished by primary teeth displaying a grayish or brownish opalescent hue, despite retaining normal morphological characteristics, although instances of premature abnormal wear have been reported. Following the eruption of primary dentition, abnormal dentin formation results in the obliteration of pulp chambers and root canals. Clinically, permanent teeth typically present with a normal appearance, while radiographically, unusually large pulp chambers are evident [12].

Differential diagnoses encompass a range of conditions, including dentinogenesis imperfecta, Kostmann syndrome, cyclic neutropenia, Chediak-Higashi syndrome, Langerhans cell histiocytosis, Papillon-Lefevre syndrome, hypophosphatasia, vitamin D-resistant rickets, amelogenesis imperfecta, and Ehlers-Danlos syndrome [13].

## Conclusions

In conclusion, DD1 presents as a rare autosomal dominant disorder characterized by structural dentin abnormalities, resulting in significant dental issues. Clinical manifestations include generalized slight yellowish discoloration and tooth mobility, while radiographic examination often reveals reduced pulp chambers without pulp stones, a defining feature of DD1. Treatment necessitates a multidisciplinary approach, involving tooth extraction, bilateral direct sinus lift procedures, implant placement, and subsequent fixed prosthesis placement. Recent case studies underscore the efficacy of such interventions, showcasing improved oral health-related quality of life, functional restoration, and enhanced esthetics. Early diagnosis and intervention are pivotal in managing DD1, highlighting the importance of a multidisciplinary approach in addressing its complex dental challenges. Further research is warranted to advance our understanding of DD1's genetic basis and develop targeted therapies for optimized patient outcomes.
